# Epidemiological Transition of Nonalcoholic Fatty Liver Disease and Cirrhosis in Asia: Three Decades of Spatiotemporal Dynamics, Health Inequality, and Future Burden

**DOI:** 10.1155/cjgh/4834539

**Published:** 2026-07-13

**Authors:** Xiaoheng Liu, Chen Zhou

**Affiliations:** ^1^ Department of General Internal Medicine, Sichuan Integrative Medicine Hospital, Chengdu, Sichuan, China, scu.edu.cn

**Keywords:** Asian trends, disease burden, health inequalities, multidimensional analysis, NAFLD, predictive modeling

## Abstract

**Objective:**

To analyze spatiotemporal distribution, health inequalities, and future trends of nonalcoholic fatty liver disease (NAFLD) burden in Asia from 1990 to 2023.

**Methods:**

Using Global Burden of Disease (GBD) 2023 data, age‐standardized prevalence rates (ASPRs), incidence rates (ASIR), mortality rates (ASMR), and disability‐adjusted life years (DALYs) rates (ASDR) were calculated. Joinpoint regression analyzed temporal trends, age‐period‐cohort models evaluated multidimensional effects, and Das Gupta decomposition explored contributions from population, aging, and epidemiological factors. Data envelopment analysis (DEA) assessed relationships with Human Development Index (HDI), while slope index of inequality (SII) and concentration index (CI) analyzed health inequalities. Bayesian age‐period‐cohort (BAPC) model projected 2024–2038 trends.

**Results:**

In 2023, Asia carried 797.20 million prevalent cases (95% UI 693.74–910.71), 30.61 million incident cases, 27,083 deaths, and 760,383 DALYs. ASPR and ASIR had increased since 1990 (EAPC +0.851% and +0.696%), while ASMR (−0.903%) and ASDR (−0.895%) had fallen. Country dispersion was extreme: Kuwait’s ASPR exceeded Japan’s more than fourfold, and Central Asia’s ASMR was an order of magnitude above East Asia’s. Decomposition attributed 69.75% of prevalence‐burden change to population growth and 29.00% to epidemiologic factors; East Asia was uniquely dominated by aging (47.24%). DEA flagged efficiency gaps in several upper‐HDI states, including Kazakhstan and the Gulf countries. SII rose from 4.882 to 8.864 (81.6%), while CI remained negative throughout, signaling widening absolute inequality and persistent concentration of burden among lower‐HDI populations. Projections to 2038 suggested gradual decline in all four indicators.

**Conclusion:**

Asia’s NAFLD trajectory is not a uniform rise. Morbidity is expanding while prognosis improves, and efficiency and equity gaps are growing. One‐size‐fits‐all continental strategies will underdeliver; development‐stage‐specific responses are required.

## 1. Introduction

Nonalcoholic fatty liver disease (NAFLD) is now the most common chronic liver condition globally. It is defined by hepatic steatosis affecting at least 5% of hepatocytes in the absence of competing etiologies such as significant alcohol intake or chronic viral hepatitis [1, 2]. Pooled prevalence estimates in adults are close to 30% [3, 4], and the disease spectrum from simple steatosis through steatohepatitis, fibrosis, cirrhosis, and hepatocellular carcinoma has been recognized to intersect cardiometabolic, renal, and oncologic morbidity [5–8].

Asia merits separate analysis for reasons that are, in our view, stronger than typical continental burden papers acknowledged. Body composition and metabolic susceptibility differ from Western reference populations: visceral adiposity and insulin resistance manifest at lower body mass indices [9, 10], and the PNPLA3 I148M risk allele segregates at higher frequency in several East and South Asian populations [11–13], both of which shift the dose–response between adiposity and hepatic disease. The pace of nutritional transition, urbanization, and access to noninvasive diagnostic technologies (transient elastography, MR‐based fat quantification, and validated serum panels) has been unusually compressed across the region [14–16], creating overlapping cohorts that experience markedly different lifetime exposures. The continent also spans the full human‐development continuum, from low‐income settings in which underdetection probably dominates to high‐income city‐states in which prognostic gains may be approaching their natural ceiling. A continental analysis, therefore, captures variation that within‐country or within‐subregion studies cannot.

The methodological motivation is more specific. Most published GBD‐based NAFLD analyses describe rates and trends [16, 17]. Comparatively few attempts were done to attribute observed changes to demographic versus epidemiologic forces and fewer still benchmark country‐level performance against what would be achievable given a country’s development level. We argue that combining these complementary lenses (decomposition for attribution, DEA for efficiency, SII and CI for equity, and Bayesian age‐period‐cohort (BAPC) for projection) yields qualitatively different inferences than any single technique applied alone. Two countries with identical 2023 ASDR may differ in whether their burden is rising because of population growth or because of the underlying risk‐factor expansion, and whether their burden is high relative to peers at the same HDI. These distinctions matter for policy.

We, therefore, conducted a continent‐wide assessment of NAFLD (cirrhosis‐inclusive) across 49 Asian countries from 1990 through 2023, with three prespecified objectives (i) to quantify spatiotemporal heterogeneity in prevalence, incidence, mortality, and DALY rates; (ii) to attribute a three‐decade burden change to demographic growth, population aging, and epidemiologic risk and to benchmark country‐level efficiency and inequality against development‐adjusted expectations; and (iii) to project the continental trajectory to 2038 under a continuation‐of‐trend assumption while making the limits of long‐horizon projection explicit. The aim, beyond updating estimates, is to demonstrate how a layered analytic approach can move continental burden research from description toward prioritization.

Relative to the existing Asia‐focused NAFLD literature [14–17], this paper makes four contributions. First, it draws on the most recent GBD 2023 release with a country‐specific 1990 to 2023 series [26, 49], extending earlier syntheses that ended in 2017 or 2019. Second, it operationalizes the DEA‐HDI efficiency frontier as an explicit accountability tool, separating countries with genuinely high burden from those whose burden is high relative to their development level, a distinction of direct relevance to ministries of health. Third, it quantifies trends in absolute (SII) and relative (CI) inequality jointly, showing that the two need not move in the same direction. Fourth, it documents an Asia‐specific transition signature in which prognosis indicators are improving while exposure indicators remain elevated, an asymmetry that is rarely highlighted in burden papers and that has implications for resource allocation.

## 2. Materials and Methods

### 2.1. Data Source, Case Definition, and Population Coverage

Estimates were obtained from the Global Burden of Disease Study, accessed through the Global Health Data Exchange query interface. The case definition follows the GBD modeling group: NAFLD captured as a chronic‐liver‐disease cause with cirrhosis as the modeled severe outcome, ascertained through triangulation of vital registration, hospital discharge, claims, survey, and imaging‐based sources, with DisMod‐MR 2.1 used to enforce internal consistency between prevalence, incidence, remission, and excess mortality. Four standardized metrics (per 100,000 population), including age‐standardized prevalence rate (ASPR), age‐standardized incidence rate (ASIR), age‐standardized mortality rate (ASMR), and age‐standardized DALYs rate (ASDR), were extracted together with corresponding case counts and 95% uncertainty intervals (UIs). The geographic frame comprised 49 Asian countries and territories, grouped into five subregions for stratified analysis (East, Southeast, South, Central Asia, with Western Asian states reported within country tables). Sex‐ and age‐specific rates were obtained in 5‐year bands.

### 2.2. Analytical Strategy and Rationale for Each Method

The analysis was layered, and each method was chosen to answer a question the others could not. Reviewer feedback specifically asked us to justify rather than enumerate methods, and we therefore make the inferential mapping explicit before describing technical implementation.

#### 2.2.1. Joinpoint Regression: Locating Inflection Points

Joinpoint regression was used not as a duplicate of EAPC estimation but to detect the timing of statistically significant changes in trend slope, which an EAPC summary necessarily averages over. We fit log‐linear segmented models on annual age‐standardized rates with up to five permitted joinpoints, evaluated with a Monte Carlo permutation test at the 0.05 level, and computed the annual percentage change (APC) within each segment. The substantive output is the calendar year(s) at which the trajectory bent, which matters when aligning epidemiologic dynamics with policy or therapeutic milestones.

#### 2.2.2. Age‐Period‐Cohort Modeling: Separating Biologic Age From Secular and Generational Forces

Because age, period, and cohort are linearly dependent, conventional EAPC and joinpoint outputs cannot distinguish whether a rising rate reflects an aging population, a calendar‐time shift in risk such as diagnostic modernization, or a generational change in lifetime exposure. We implemented an APC framework using the U.S. National Cancer Institute’s estimable‐functions parameterization, restricting the panel to 1994 to 2023 in 5‐year periods (1990–1993 excluded to preserve grid alignment). The APC analysis returns three substantively distinct quantities: longitudinal age curves (net of period and cohort), period rate ratios with 2006 as referent, and cohort rate ratios with the 1959 birth cohort as referent. Local drift, the age‐specific annualized change, is reported additionally because divergence between local and net drift is the most informative signal that birth‐cohort effects are driving observed trends.

#### 2.2.3. Das Gupta Decomposition: Attributing 1990–2023 Change

To partition observed change in prevalent cases, incident cases, deaths, and DALYs between (i) population growth, (ii) shifts in population age structure, and (iii) age‐specific epidemiologic rates, we applied Das Gupta’s standardization‐based decomposition. The method is preferred over Kitagawa‐style two‐way decompositions for three‐factor problems because it is symmetric, that is, results do not depend on the order in which factors are introduced, and because contributions sum exactly to the observed difference. Decomposition was performed for the continent overall, by sex, and for the four GBD Asian subregions; absolute contributions and percentages of the total change are reported.

#### 2.2.4. DEA Frontier Against HDI: A Development‐Adjusted Efficiency Benchmark

Standard burden papers report whether a country’s rate is high or low. They rarely answer the more policy‐relevant question of whether a country’s rate is high or low relative to what is achievable at its level of development. We constructed a nonparametric production frontier using a free‐disposal hull (FDH) data envelopment analysis specification, with HDI as the input and ASDR as the (undesirable) output, restricted to the 32 Asian countries with continuous HDI series 1990–2023. A locally weighted scatterplot smoother (LOWESS, span 0.5) was overlaid for visualization. Super‐efficient observations beneath the frontier (data anomalies) were trimmed before fitting. The vertical distance between an observed country‐year and the frontier represents the avoidable‐burden component, the fraction of ASDR not explained by HDI alone, and constitutes our country‐level efficiency metric.

#### 2.2.5. Slope Index of Inequality and Concentration Index: Equity Over Time

Health equity was assessed in two complementary metrics. The slope index of inequality (SII) regresses the DALY rate on a population‐weighted rigid transformation of HDI rank and reports absolute inequality in the original outcome scale. The concentration index (CI) is twice the area between the concentration curve and the line of equality and reports relative inequality bounded in [‐1, 1], with negative values indicating concentration of burden among lower HDI populations. Reporting both is important because absolute and relative inequality can move in opposite directions, a phenomenon we observed in our data.

#### 2.2.6. BAPC Projection

Forward projections to 2038 used the BAPC framework with second‐order random‐walk priors on age, period, and cohort effects, integrated nested Laplace approximation (INLA) for posterior inference, and overdispersed Poisson likelihood for counts. BAPC was selected over autoregressive alternatives because it propagates uncertainty in age, period, and cohort components separately, producing wider but more honest predictive intervals. Point predictions and 95% credible intervals are reported, and we caution against literal interpretation of the upper‐horizon estimates, which inherit the assumption that 1990–2023 trends will continue.

### 2.3. Software, Reproducibility, and Reporting

All analyses were executed in R 4.2.0. Joinpoint regression used the U.S. National Cancer Institute’s Joinpoint Regression Program (v5.0). Age‐period‐cohort analyses used the NCI online APC Web Tool and were independently replicated in R using the apc package. Decomposition, DEA, SII/CI, and BAPC analyses used purpose‐built R routines based on standard packages (segmented, deaR, INLA). Two‐sided *p* < 0.05 was treated as statistically significant. Continental estimates and percentages presented in the text are rounded for readability; precise values, 95% UIs, and EAPC estimates appear in Tables 1 and 2.

**TABLE 1:** NAFLD prevalent cases, incident cases, age‐standardized prevalence, and incidence rates in Asia, its subregions, and 49 Asian countries (regions) in 2023, with estimated annual percentage changes from 1990 to 2023.

Location	Prevalence number (95% UI)	ASPR (95% UI)	ASPR EAPC 1990–2023 (95% UI)	Incidence number (95% UI)	ASIR (95% UI)	ASIR EAPC 1990–2023 (95% UI)
Asia	797196647.587 (693739137.808–910713400.865)	14965.250 (12996.809–17125.760)	0.851 (0.766–0.936)	30611152.558 (21045865.492–42108879.365)	607.867 (420.442–831.708)	0.696 (0.632–0.761)
East Asia	298477686.259 (273572421.097–328604795.607)	15041.436 (13771.289–16655.683)	0.884 (0.711–1.058)	9690713.628 (8900077.357–10503169.277)	604.089 (547.331–654.394)	0.745 (0.586–0.904)
Southeast Asia	115675312.476 (105598596.170–128497844.945)	15116.226 (13794.431–16757.732)	0.561 (0.534–0.589)	4662839.734 (4255665.576–5040566.388)	613.156 (560.752–664.529)	0.458 (0.435–0.480)
Central Asia	14921486.106 (13579231.451–16588253.055)	15200.519 (13856.724–16840.178)	0.615 (0.565–0.664)	591144.410 (542247.356–642351.771)	604.844 (556.696–656.852)	0.534 (0.502–0.567)
South Asia	230776079.019 (209604217.249–257699345.949)	12792.942 (11650.473–14227.005)	0.701 (0.632–0.769)	10827279.914 (9875425.242–11894302.735)	552.813 (505.480–601.476)	0.605 (0.543–0.667)
Pakistan	26263490.489 (23782361.078–29397118.509)	14549.311 (13269.511–16226.384)	0.693 (0.675–0.710)	1342959.004 (1222624.559–1483326.552)	619.666 (570.515–672.923)	0.593 (0.575–0.612)
Nepal	3402705.092 (3100662.796–3802091.340)	11559.850 (10558.101–12852.980)	0.903 (0.843–0.963)	158680.903 (145183.166–174398.780)	494.271 (452.391–539.703)	0.876 (0.814–0.938)
India	180038445.644 (163380111.619–201066007.041)	12554.497 (11418.520–13986.760)	0.695 (0.611–0.779)	8336830.114 (7590532.507–9152774.653)	544.162 (496.093–592.863)	0.593 (0.517–0.669)
Bhutan	108548.119 (98210.178–121233.233)	14075.279 (12839.116–15571.549)	0.909 (0.879–0.939)	4781.863 (4339.486–5273.789)	586.505 (535.884–639.068)	0.761 (0.734–0.789)
Bangladesh	20962889.674 (19029523.052–23472472.223)	13180.366 (11986.863–14711.812)	0.696 (0.672–0.720)	984028.030 (893200.605–1083319.104)	554.845 (506.361–602.236)	0.594 (0.569–0.619)
Afghanistan	5103035.312 (4586623.964–5712509.977)	23119.066 (21018.338–25409.713)	0.711 (0.657–0.766)	277658.562 (246998.777–305623.504)	916.981 (838.706–993.583)	0.630 (0.604–0.657)
Yemen	5390703.723 (4864130.039–5991506.011)	21681.858 (19712.934–23875.709)	0.630 (0.606–0.653)	273950.704 (245409.248–304500.110)	863.436 (788.565–937.149)	0.564 (0.546–0.582)
United Arab Emirates	4318115.611 (3931087.676–4761162.194)	32428.584 (29959.130–35194.483)	0.980 (0.947–1.014)	103268.374 (91510.616–118887.739)	1084.697 (1011.841–1150.146)	0.724 (0.673–0.776)
Turkey	26930189.244 (24644349.267–29392242.067)	27361.821 (24986.144–29953.387)	0.829 (0.801–0.858)	868168.863 (809089.203–924043.490)	952.159 (884.380–1020.044)	0.675 (0.653–0.697)
Syrian Arab Republic	4998945.561 (4569517.745–5511395.849)	28857.763 (26483.359–31763.992)	0.754 (0.727–0.780)	201707.584 (184099.576–217862.564)	1050.831 (965.721–1125.124)	0.638 (0.614–0.663)
Saudi Arabia	10925281.227 (9809387.656–12117982.570)	32903.122 (30151.908–35978.101)	1.087 (1.053–1.120)	411991.400 (376507.140–450347.416)	1118.462 (1041.981–1193.181)	0.809 (0.775–0.842)
Qatar	1282074.294 (1160004.118–1410661.835)	34859.715 (32081.216–37732.840)	0.882 (0.845–0.920)	39074.714 (34923.357–43982.821)	1135.870 (1061.077–1201.134)	0.657 (0.625–0.690)
Oman	1592682.143 (1424248.016–1774900.240)	30723.485 (28088.097–33838.126)	^∗^1.220 (1.179–1.261)	57512.590 (52237.318–63313.224)	1071.585 (997.455–1140.243)	^∗^0.952 (0.913–0.990)
Palestine	1186051.764 (1079486.742–1311982.985)	27819.399 (25535.941–30429.667)	0.693 (0.670–0.716)	53586.500 (48523.864–58439.713)	1003.063 (920.824–1074.710)	0.576 (0.554–0.597)
Lebanon	1781707.413 (1631045.477–1947764.417)	28937.310 (26545.164–31633.240)	0.798 (0.779–0.816)	60425.878 (55643.830–65263.973)	1030.512 (951.410–1105.522)	0.669 (0.653–0.686)
Kuwait	1911196.486 (1746852.613–2098322.229)	^∗^35363.464 (32631.581–38514.666)	0.850 (0.812–0.887)	62479.502 (57920.084–67140.791)	^∗^1174.264 (1096.666–1245.335)	0.662 (0.624–0.700)
Jordan	3867351.599 (3513015.428–4271674.638)	31211.599 (28520.252–34145.838)	0.956 (0.925–0.987)	154293.693 (141007.881–166272.827)	1080.456 (997.140–1152.235)	0.728 (0.701–0.755)
Iraq	10370914.675 (9420576.446–11449772.368)	27875.131 (25670.028–30579.062)	0.651 (0.610–0.693)	453069.611 (412412.022–492682.471)	1012.194 (931.753–1088.060)	0.560 (0.527–0.594)
Islamic Republic of Iran	31210531.028 (28412442.876–34250217.505)	31829.771 (29183.770–34811.413)	1.084 (0.893–1.275)	1052260.618 (983881.714–1129943.814)	1150.996 (1067.573–1227.085)	0.874 (0.723–1.024)
Bahrain	573198.396 (522744.087–627266.038)	32038.150 (29492.842–34842.342)	0.855 (0.830–0.880)	18870.276 (17469.887–20326.621)	1078.675 (1000.194–1145.184)	0.651 (0.625–0.678)
Israel	1434892.643 (1307668.972–1585863.827)	13389.807 (12196.558–14822.570)	0.695 (0.644–0.746)	48353.521 (44702.787–52523.765)	483.838 (442.941–525.006)	0.637 (0.586–0.688)
Cyprus	186015.804 (168355.384–205992.625)	10474.142 (9469.276–11614.078)	0.683 (0.626–0.740)	5829.268 (5307.732–6353.826)	392.501 (354.515–427.179)	0.621 (0.569–0.672)
Singapore	1004695.427 (918128.494–1112011.202)	12730.528 (11655.111–14142.064)	0.459 (0.423–0.494)	29167.135 (26475.558–31975.687)	479.883 (437.603–524.608)	0.369 (0.334–0.404)
Republic of Korea	7517172.849 (6881479.887–8268211.396)	9900.308 (9013.028–10988.372)	0.887 (0.580–1.195)	227042.562 (206863.848–247712.714)	385.157 (351.215–421.008)	0.748 (0.495–1.002)
Japan	16109735.820 (14725867.407–17638177.908)	^∗^7838.962 (7192.796–8697.921)	^∗^0.293 (0.234–0.351)	452763.840 (410682.586–491202.426)	^∗^319.531 (293.628–347.353)	^∗^0.254 (0.196–0.311)
Brunei Darussalam	^∗^62914.281 (56527.099–70151.833)	12267.246 (11143.514–13708.020)	0.448 (0.380–0.516)	^∗^2415.580 (2211.658–2647.018)	480.103 (441.081–522.412)	0.435 (0.347–0.522)
Uzbekistan	5256739.775 (4756846.539–5863154.897)	15763.344 (14283.248–17493.696)	0.647 (0.593–0.702)	215688.025 (196817.052–234793.829)	626.167 (573.093–680.615)	0.551 (0.514–0.588)
Turkmenistan	816085.866 (741492.173–906643.964)	15650.466 (14334.550–17311.657)	0.764 (0.704–0.824)	35193.556 (32284.603–38564.493)	629.316 (580.880–686.133)	0.718 (0.678–0.758)
Tajikistan	1157988.270 (1049074.356–1294053.618)	13708.121 (12422.634–15207.940)	0.376 (0.319–0.433)	55778.313 (50513.682–61437.366)	563.300 (513.822–613.606)	0.297 (0.262–0.332)
Mongolia	399165.905 (363212.823–445937.437)	12689.276 (11580.805–14080.229)	0.495 (0.405–0.585)	16814.673 (15345.916–18532.305)	520.953 (479.317–568.287)	0.410 (0.345–0.475)
Kyrgyzstan	961576.521 (872235.300–1074143.413)	14790.010 (13471.752–16430.681)	0.371 (0.339–0.404)	42009.502 (38302.231–46177.085)	603.321 (551.754–658.295)	0.297 (0.274–0.321)
Kazakhstan	3009461.559 (2722628.435–3361547.982)	14432.624 (13053.467–16116.182)	0.621 (0.550–0.692)	111700.411 (102911.137–120914.099)	573.615 (524.723–627.277)	0.590 (0.525–0.655)
Georgia	705684.933 (643947.438–778686.984)	15166.085 (13740.520–16873.809)	0.432 (0.387–0.477)	21795.846 (20077.874–23638.923)	599.735 (547.983–650.090)	0.391 (0.347–0.435)
Azerbaijan	1973149.143 (1786776.259–2196228.637)	16604.736 (15085.786–18409.477)	0.780 (0.716–0.845)	72602.471 (66468.729–78975.217)	657.669 (604.301–716.258)	0.646 (0.595–0.698)
Armenia	641634.135 (588203.553–709809.430)	16052.885 (14661.338–17826.059)	0.753 (0.713–0.794)	19561.613 (17950.909–21312.483)	624.896 (576.430–681.303)	0.606 (0.565–0.646)
Socialist Republic of Viet Nam	14277149.093 (13033077.366–15801114.897)	12484.545 (11407.609–13820.644)	0.552 (0.470–0.634)	540344.910 (494300.250–587631.576)	503.095 (457.967–545.271)	0.332 (0.271–0.392)
Timor‐Leste	150485.381 (136749.814–167518.864)	13556.438 (12335.008–15094.849)	0.306 (0.285–0.327)	7585.199 (6860.503–8335.378)	561.810 (512.879–614.143)	0.321 (0.284–0.359)
Thailand	13702061.247 (12456377.116–14987236.895)	14716.315 (13391.915–16241.558)	0.661 (0.641–0.680)	430072.189 (394187.436–464300.512)	587.402 (536.330–640.298)	0.552 (0.526–0.578)
Sri Lanka	4150458.432 (3796083.557–4604762.926)	15654.091 (14253.455–17399.430)	0.510 (0.474–0.545)	155147.025 (142306.691–168633.493)	641.605 (584.250–700.220)	0.469 (0.440–0.498)
Philippines	13453490.399 (12128478.712–15000412.132)	12898.536 (11689.170–14321.316)	0.368 (0.353–0.384)	611709.756 (554501.454–671354.560)	522.361 (477.324–571.643)	0.321 (0.304–0.338)
Myanmar	8459521.252 (7721000.380–9470002.600)	15035.839 (13716.982–16741.180)	0.499 (0.423–0.575)	369422.576 (337969.624–399644.147)	623.912 (571.389–673.248)	0.407 (0.345–0.468)
Maldives	90789.735 (81831.206–101688.333)	15895.183 (14527.981–17606.576)	0.656 (0.614–0.697)	3890.561 (3515.071–4280.193)	622.179 (568.266–679.973)	0.497 (0.456–0.538)
Malaysia	7202573.056 (6520481.451–7985325.647)	19656.987 (17834.590–21709.159)	0.579 (0.553–0.606)	286998.863 (260178.181–313080.222)	762.237 (691.871–827.190)	0.484 (0.459–0.508)
Lao People’s Democratic Republic	746250.434 (669847.812–835693.697)	11396.488 (10396.955–12653.622)	0.513 (0.484–0.543)	34435.194 (30996.739–37962.259)	457.738 (416.570–498.186)	0.427 (0.394–0.460)
Indonesia	51106714.595 (46539808.364–57059253.691)	16715.733 (15281.193–18562.332)	0.621 (0.596–0.645)	2123795.314 (1943983.465–2309127.133)	683.208 (627.616–742.231)	0.531 (0.508–0.554)
Cambodia	1924497.277 (1731090.970–2152178.386)	11934.613 (10772.761–13276.398)	0.387 (0.303–0.470)	84722.244 (77291.517–92770.824)	489.794 (447.318–531.609)	0.309 (0.234–0.384)
Taiwan (Province of China)	5530030.800 (5082813.428–6099329.605)	16300.175 (14825.592–18059.567)	0.804 (0.720–0.887)	163772.890 (150225.125–177451.360)	628.195 (574.016–682.241)	0.769 (0.697–0.841)
Democratic People’s Republic of Korea	4311881.010 (3915353.486–4768053.553)	13278.903 (12061.814–14748.322)	0.482 (0.466–0.497)	161828.983 (147663.280–177264.088)	542.365 (495.689–593.649)	0.449 (0.433–0.465)
China	^∗^288635774.449 (264471295.956–317923089.040)	15052.219 (13777.629–16667.789)	0.893 (0.714–1.071)	^∗^9365111.755 (8596338.540–10153431.050)	605.134 (548.265–655.537)	0.752 (0.587–0.916)

^∗^The highest and lowest values within each column across all countries/locations, excluding the aggregate estimate for the “Asia” region.

**TABLE 2:** NAFLD deaths, DALYs, age‐standardized mortality and DALYs rates in Asia, its subregions, and 49 Asian countries (regions) in 2023, with estimated annual percentage changes from 1990 to 2023.

Location	Death number (95% UI)	ASMR (95% UI)	ASMR EAPC 1990–2023 (95% UI)	DALYs number (95% UI)	ASDR (95% UI)	ASDR EAPC 1990–2023 (95% UI)
Asia	27083.401 (13106.836–48220.822)	0.511 (0.247–0.912)	−0.903 (−0.960–−0.846)	760383.191 (365181.533–1372053.676)	13.989 (6.709–25.287)	−0.895 (−0.953–−0.837)
East Asia	5248.654 (3473.475–7539.950)	0.227 (0.154–0.319)	−2.292 (−2.401–−2.183)	132368.340 (87084.838–193186.241)	5.815 (3.913–8.198)	−2.403 (−2.510–−2.296)
Southeast Asia	7021.220 (4610.945–9767.620)	0.986 (0.656–1.366)	0.350 (0.261–0.439)	218765.847 (146166.496–301505.923)	28.661 (19.457–38.821)	0.396 (0.319–0.473)
Central Asia	2409.505 (1658.245–3299.948)	2.787 (1.941–3.768)	2.005 (1.735–2.276)	72571.013 (48372.608–101412.787)	75.591 (51.910–103.228)	1.889 (1.611–2.167)
South Asia	7135.206 (4703.637–10166.921)	0.463 (0.306–0.655)	−0.687 (−0.946–−0.428)	225143.516 (147826.873–319386.144)	13.142 (8.800–18.654)	−1.005 (−1.258–−0.752)
Pakistan	803.322 (448.793–1214.230)	0.543 (0.313–0.810)	−0.299 (−0.562–−0.035)	28203.684 (16105.722–42069.536)	16.580 (9.477–24.867)	−0.373 (−0.622–−0.123)
Nepal	151.527 (77.229–236.927)	0.581 (0.296–0.902)	1.113 (0.990–1.235)	4927.234 (2645.535–7535.853)	17.239 (9.102–26.591)	0.875 (0.757–0.993)
India	^∗^5888.223 (3892.848–8437.104)	0.475 (0.309–0.666)	−0.577 (−0.836–−0.318)	^∗^181830.436 (120871.283–258869.212)	13.300 (8.875–18.904)	−0.907 (−1.177–−0.637)
Bhutan	6.890 (3.769–11.386)	1.022 (0.546–1.702)	1.039 (0.804–1.273)	218.114 (117.359–350.579)	29.673 (15.804–47.284)	0.729 (0.489–0.970)
Bangladesh	285.244 (167.511–592.265)	0.223 (0.132–0.487)	−3.236 (−3.756–−2.714)	9964.048 (5801.431–17759.356)	6.553 (3.892–12.275)	−3.829 (−4.190–−3.466)
Afghanistan	31.313 (17.623–50.954)	0.291 (0.155–0.468)	−0.330 (−0.492–−0.167)	996.816 (564.786–1646.924)	7.044 (4.027–11.060)	−0.290 (−0.453–−0.126)
Yemen	67.485 (36.503–108.914)	0.540 (0.291–0.889)	−1.270 (−1.421–−1.118)	1752.658 (958.131–2653.425)	11.186 (6.048–17.468)	−1.324 (−1.450–−1.197)
United Arab Emirates	49.468 (31.588–71.859)	2.767 (1.469–4.192)	0.169 (−0.098–0.437)	1351.875 (853.969–1974.323)	40.001 (24.654–57.810)	−0.580 (−0.852–−0.308)
Turkey	298.470 (174.280–466.827)	0.326 (0.189–0.510)	0.147 (−0.249–0.546)	6093.878 (3643.471–9417.778)	6.247 (3.834–9.545)	−0.254 (−0.597–0.090)
Syrian Arab Republic	118.469 (72.404–177.044)	1.110 (0.683–1.692)	0.969 (0.735–1.204)	2704.246 (1734.444–4143.973)	19.802 (12.716–28.748)	0.572 (0.328–0.816)
Saudi Arabia	215.384 (140.938–308.895)	2.146 (1.314–3.295)	1.975 (1.563–2.387)	5936.696 (3921.308–8315.522)	38.987 (24.873–56.497)	1.637 (1.233–2.044)
Qatar	9.929 (6.467–14.279)	1.988 (1.123–3.019)	−1.728 (−2.330–−1.121)	286.784 (175.375–411.636)	32.446 (20.115–47.266)	−1.999 (−2.602–−1.392)
Oman	25.714 (15.239–39.264)	1.628 (0.883–2.569)	3.545 (3.260–3.831)	660.058 (410.418–1026.067)	30.202 (17.682–46.382)	2.930 (2.665–3.196)
Palestine	11.616 (7.141–16.793)	0.517 (0.312–0.780)	−1.387 (−1.555–−1.219)	274.263 (170.897–404.509)	10.159 (6.373–14.832)	−1.604 (−1.750–−1.457)
Lebanon	46.273 (27.728–69.950)	0.746 (0.442–1.111)	−0.655 (−1.031–−0.278)	971.076 (598.339–1388.717)	16.197 (10.011–23.314)	−0.535 (−0.961–−0.107)
Kuwait	13.851 (8.924–19.173)	0.783 (0.506–1.155)	0.432 (−0.525–1.397)	371.353 (241.332–507.533)	13.955 (9.228–19.992)	0.055 (−0.909–1.029)
Jordan	47.659 (29.879–66.893)	0.691 (0.433–0.964)	−1.158 (−1.458–−0.857)	1156.635 (749.565–1642.496)	13.724 (8.602–18.883)	−1.323 (−1.629–−1.016)
Iraq	170.629 (104.134–265.979)	0.800 (0.476–1.259)	−0.057 (−0.310–0.198)	4807.581 (2879.558–7536.021)	17.613 (10.969–27.221)	−0.423 (−0.663–−0.183)
Islamic Republic of Iran	1420.114 (887.473–2184.322)	2.044 (1.204–3.222)	−0.836 (−1.084–−0.589)	33731.272 (22800.338–50800.254)	41.260 (27.145–62.049)	−1.225 (−1.453–−0.997)
Bahrain	10.971 (6.794–16.603)	1.901 (1.057–3.055)	−0.388 (−0.662–−0.113)	277.486 (173.148–435.539)	31.902 (19.053–48.929)	−0.846 (−1.113–−0.578)
Israel	103.827 (75.568–136.013)	0.814 (0.594–1.072)	−1.454 (−1.778–−1.128)	2380.200 (1736.708–3163.070)	20.363 (14.796–27.212)	−1.425 (−1.746–−1.104)
Cyprus	17.083 (10.811–24.147)	0.882 (0.560–1.243)	−1.386 (−1.463–−1.310)	411.861 (260.462–569.424)	21.533 (13.618–29.617)	−1.450 (−1.528–−1.372)
Singapore	15.289 (10.279–22.904)	^∗^0.167 (0.113–0.247)	−2.418 (−2.555–−2.280)	347.060 (235.137–522.674)	^∗^3.816 (2.615–5.652)	−2.870 (−3.015–−2.724)
Republic of Korea	379.648 (253.740–532.290)	0.395 (0.268–0.554)	^∗^‐4.689 (−4.939–−4.438)	8594.200 (5772.655–12005.487)	9.454 (6.407–13.333)	^∗^‐4.768 (−4.965–−4.571)
Japan	2237.949 (1537.374–3160.087)	0.554 (0.397–0.755)	−1.845 (−2.027–−1.662)	39094.880 (27904.372–53043.307)	13.261 (9.278–18.174)	−1.992 (−2.168–−1.816)
Brunei Darussalam	1.728 (1.071–2.559)	0.402 (0.251–0.580)	−0.682 (−0.751–−0.612)	53.321 (31.915–81.272)	10.868 (6.795–16.246)	−0.779 (−0.877–−0.680)
Uzbekistan	662.095 (439.597–926.390)	2.297 (1.561–3.174)	−0.284 (−0.435–−0.134)	22280.300 (14496.059–31417.949)	68.405 (45.707–93.664)	−0.093 (−0.266–0.080)
Turkmenistan	251.519 (168.282–364.398)	^∗^5.268 (3.658–7.303)	3.846 (3.561–4.131)	9082.352 (6047.798–13010.696)	^∗^173.495 (117.339–245.557)	4.355 (4.060–4.651)
Tajikistan	76.920 (51.011–110.766)	1.332 (0.857–2.003)	−0.458 (−0.774–−0.141)	2478.573 (1621.554–3499.949)	34.548 (23.093–49.824)	−0.558 (−0.895–−0.219)
Mongolia	35.939 (22.394–51.012)	1.472 (0.918–2.076)	−0.609 (−1.190–−0.024)	1170.370 (742.257–1713.142)	39.466 (24.951–55.445)	−0.702 (−1.206–−0.196)
Kyrgyzstan	103.729 (69.566–149.120)	1.936 (1.367–2.659)	−0.193 (−0.675–0.291)	3444.258 (2205.955–5131.982)	55.329 (36.706–78.213)	−0.198 (−0.765–0.372)
Kazakhstan	848.634 (583.262–1173.339)	4.486 (3.161–6.117)	^∗^5.790 (5.220–6.362)	22724.090 (15640.893–31394.301)	108.695 (76.597–148.207)	^∗^5.187 (4.565–5.813)
Georgia	80.728 (54.388–115.667)	1.454 (0.999–2.077)	0.805 (0.450–1.162)	2211.358 (1472.339–3273.211)	43.615 (28.699–63.370)	1.129 (0.739–1.521)
Azerbaijan	300.186 (194.606–431.016)	2.933 (2.009–4.266)	0.922 (0.756–1.089)	8083.148 (5126.884–11814.084)	70.196 (46.570–99.815)	0.780 (0.615–0.944)
Armenia	49.755 (35.983–67.870)	1.005 (0.726–1.345)	0.868 (−0.091–1.837)	1096.566 (772.288–1483.302)	24.590 (17.029–33.361)	0.879 (0.057–1.707)
Socialist Republic of Viet Nam	494.305 (301.614–749.139)	0.444 (0.271–0.678)	−0.267 (−0.421–−0.113)	13612.959 (8666.214–21053.333)	11.756 (7.567–17.855)	−0.155 (−0.313–0.003)
Timor‐Leste	4.319 (2.279–7.103)	0.485 (0.251–0.789)	−0.811 (−0.964–−0.659)	126.013 (68.057–200.300)	12.944 (6.991–21.231)	−0.929 (−1.103–−0.755)
Thailand	1323.773 (876.843–1906.665)	1.191 (0.803–1.676)	1.655 (1.404–1.906)	36062.750 (23680.856–51689.903)	34.699 (23.065–49.333)	1.933 (1.724–2.143)
Sri Lanka	140.103 (90.326–205.488)	0.476 (0.310–0.692)	−1.811 (−2.072–−1.548)	3511.335 (2246.326–5258.159)	12.109 (7.898–18.134)	−2.481 (−2.831–−2.130)
Philippines	422.926 (274.316–623.007)	0.498 (0.322–0.719)	0.328 (0.241–0.415)	12910.287 (8473.007–18842.285)	13.440 (8.714–19.250)	0.231 (0.151–0.312)
Myanmar	371.875 (200.604–615.827)	0.679 (0.370–1.103)	0.223 (0.104–0.341)	14439.321 (7616.075–25027.323)	25.116 (13.155–43.096)	0.387 (0.247–0.527)
Maldives	^∗^1.418 (0.897–2.040)	0.508 (0.302–0.782)	−0.582 (−0.767–−0.397)	^∗^37.615 (24.082–54.255)	10.364 (6.547–15.009)	−1.393 (−1.576–−1.211)
Malaysia	167.859 (107.520–259.598)	0.540 (0.348–0.829)	−0.330 (−0.600–−0.058)	4512.852 (2908.227–6885.229)	13.386 (8.686–20.499)	−0.369 (−0.560–−0.177)
Lao People’s Democratic Republic	39.887 (23.543–59.886)	0.785 (0.464–1.182)	−0.258 (−0.345–−0.172)	1256.023 (769.734–1875.626)	21.188 (12.757–31.617)	−0.466 (−0.560–−0.373)
Indonesia	3935.021 (2469.748–5625.996)	1.502 (0.954–2.101)	0.387 (0.266–0.509)	128688.631 (84208.862–179504.208)	43.357 (28.291–60.271)	0.407 (0.298–0.516)
Cambodia	95.680 (53.518–155.519)	0.699 (0.385–1.138)	−0.935 (−0.982–−0.888)	2888.338 (1617.065–4496.037)	19.079 (10.670–30.040)	−1.197 (−1.247–−1.147)
Taiwan (Province of China)	233.626 (157.462–331.125)	0.543 (0.365–0.768)	−1.033 (−1.181–−0.884)	5547.604 (3617.384–8092.954)	13.985 (9.293–20.439)	−0.930 (−1.124–−0.736)
Democratic People’s Republic of Korea	76.061 (40.663–124.430)	0.237 (0.127–0.383)	−0.904 (−0.992–−0.816)	2186.984 (1154.226–3604.324)	6.418 (3.554–10.642)	−0.853 (−0.934–−0.771)
China	4938.968 (3260.110–7138.611)	0.220 (0.148–0.313)	−2.366 (−2.483–−2.249)	124633.752 (81959.334–182426.235)	5.652 (3.790–7.989)	−2.485 (−2.599–−2.371)

^∗^The highest and lowest values within each column across all countries/locations, excluding the aggregate estimate for the “Asia” region.

## 3. Results

### 3.1. Overall Burden in 2023 and Temporal Trends, 1990–2023

In 2023, the continent registered 797.20 million prevalent cases (95% UI 693.74–910.71 million; ASPR 14,965.250 per 100,000), 30.61 million incident cases (ASIR 607.867 per 100,000), 27,083 deaths (ASMR 0.511 per 100,000), and 760,383 DALYs (ASDR 13.989 per 100,000) (Tables 1 and 2). Three decades of change produced a clear divergence: ASPR (EAPC +0.851%, 95% UI 0.766–0.936) and ASIR (+0.696%, 0.632–0.761) drifted upward, while ASMR (−0.903%, −0.960 to −0.846) and ASDR (−0.895%, −0.953 to −0.837) drifted downward (Tables 1 and 2).

**FIGURE 1:**
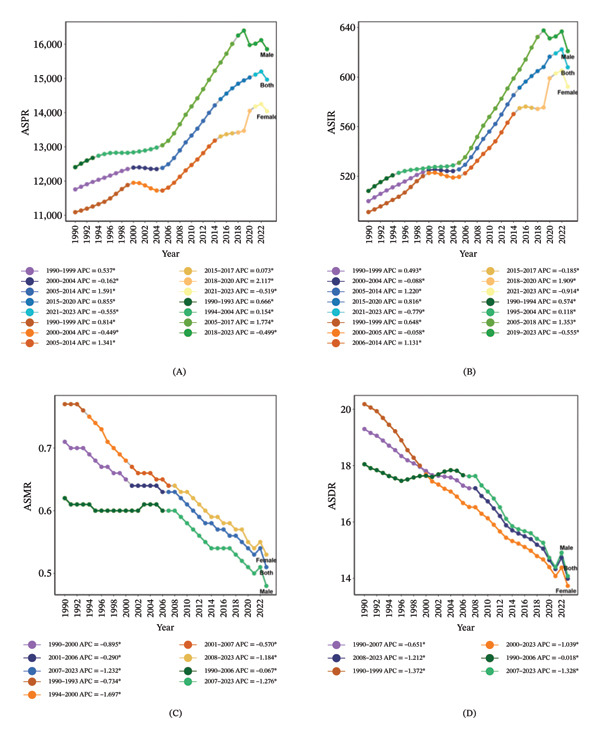
Joinpoint regression analysis of NAFLD age‐standardized rates in Asia, 1990–2023. Joinpoint regression analysis of age‐standardized (/100,000 population) prevalence (A), incidence (B), mortality (C), and DALYs rates (D) of NAFLD in Asia.

**FIGURE 2:**
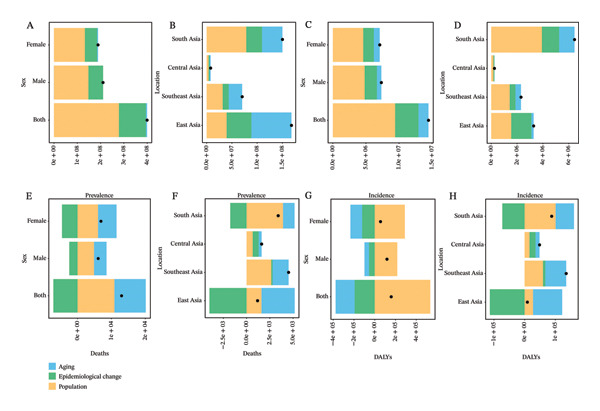
Decomposition analysis of changes in NAFLD prevalence (A and B), incidence (C and D), mortality (E and F), and DALYs (G and H) burden in Asia and four subregions from 1990 to 2023. The four Asian subregions are East Asia, Central Asia, South Asia, and Southeast Asia.

Joinpoint regression identified a nonmonotonic ASPR trajectory comprising a 1990–1999 rise (APC +0.537%), a 2000–2004 dip (−0.162%), a 2005–2014 rise (+1.591%), continued increase through 2020 (+0.855%), and a 2021–2023 reversal (−0.555%) (Figure 1A). ASIR followed a parallel structure, with the steepest rise in 2005–2014 (+1.220%) and a 2021–2023 contraction (−0.779%) (Figure 1B). ASMR declined fastest in 2007–2023 (−1.232%) and ASDR in 2008–2023 (−1.212%) (Figures 1C, 2D).

### 3.2. Country‐Level Distribution and Trend Heterogeneity

**FIGURE 3:**
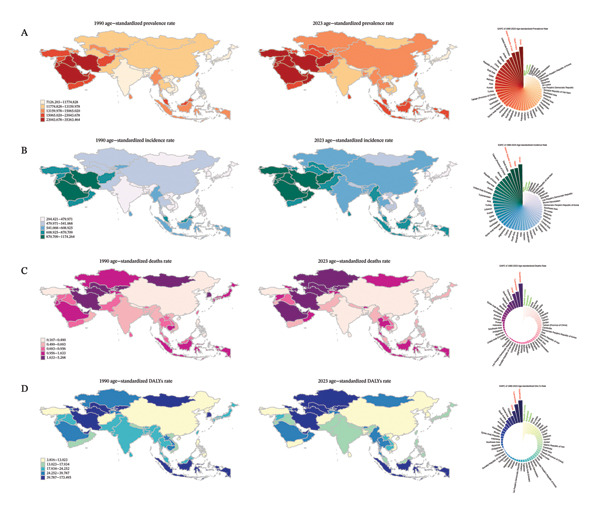
Spatial distribution and temporal trends of NAFLD disease burden in Asia (1990–2023). Distribution and changes in age‐standardized (/100,000 population) prevalence (A), incidence (B), mortality (C), and DALYs rates (D) of NAFLD in Asia. Each indicator includes three subpanels: the left panel showing the 1990 distribution, the middle panel showing the 2023 distribution, and the right panel showing the estimated annual percentage change (EAPC) from 1990 to 2023. Color intensity represents disease burden levels. In EAPC maps, red indicates the three countries with the fastest increasing burden, green indicates the three with the fastest decreasing burden.

Country‐level dispersion was substantial. ASPR ranged from 7838.962 per 100,000 in Japan to 35,363.464 per 100,000 in Kuwait, a 4.5‐fold gap (Table 1). ASMR ranged from 0.167 per 100,000 in Singapore to 5.268 per 100,000 in Turkmenistan, and ASDR from 3.816 per 100,000 in Singapore to 173.495 per 100,000 in Turkmenistan, a 45‐fold gap (Table 2). Spatial maps (Figure 3A–D) display three geographic clusters: a Central Asian cluster with high mortality (Turkmenistan, Kazakhstan, Uzbekistan, Kyrgyzstan); a Gulf cluster with high prevalence but lower mortality (Kuwait, Qatar, Saudi Arabia, United Arab Emirates, Bahrain); and an East Asian cluster with low rates but high absolute case numbers (China, Japan, Republic of Korea).

Trends differed substantially across countries (Figure 3, EAPC submaps). The fastest ASPR increases occurred in Oman (+1.220%/yr), Saudi Arabia (+1.087%/yr), and the Islamic Republic of Iran (+1.084%/yr); the slowest increase was in Japan (+0.293%/yr) (Table 1). The largest ASMR declines were observed in the Republic of Korea (−4.689%/yr), Bangladesh (−3.236%/yr), and Singapore (−2.418%/yr), whereas Kazakhstan (+5.790%/yr), Turkmenistan (+3.846%/yr), and Oman (+3.545%/yr) showed the largest mortality increases (Table 2).

### 3.3. Decomposition of Burden Change, 1990–2023

The Das Gupta decomposition apportioned the 1990–2023 change in prevalent cases as 69.75% from population growth, 29.00% from epidemiologic factors, and 1.24% from population aging (Figure 2A, B). Subregional patterns differed: in East Asia, aging contributed 47.24% of prevalence‐burden change, whereas in Southeast, Central, and South Asia, the aging share was small (< 5%) and population growth contributed 45.07%, 48.12%, and 52.21%, respectively (Figure 2A, B). Decomposition of incident cases produced a comparable continental pattern, with population growth contributing 65.09% and epidemiologic factors 24.44% (Figure 2C, D).

Mortality and DALY decompositions differed in structure (Figure 2E–H). Continental ASMR change comprised 83.51% from population growth and 71.50% from aging, both upward, offset by −55.01% from epidemiologic factors (Figure 2E, F). The negative epidemiologic contribution was larger in females (−66.77%) than in males (−40.45%) and reached −338.71% in East Asia. DALY‐burden decomposition followed a similar architecture, with population growth contributing 336.96% offset by negative aging (−115.36%) and epidemiologic (−121.60%) effects (Figure 2G, H).

### 3.4. Relationship With HDI and Health Inequality

**FIGURE 4:**
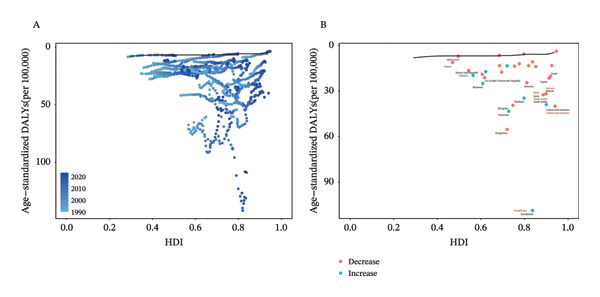
Relationship between Human Development Index and NAFLD age‐standardized DALY rate, 1990–2023. (A) Scatter plot of HDI versus NAFLD (including cirrhosis) age‐standardized DALYs rate for 32 countries from 1990 to 2023. The black curve represents the frontier production function fitting curve, indicating minimum DALYs rate at given HDI levels. Different colored points represent different years. (B) Trends in NAFLD (including cirrhosis) age‐standardized DALYs rates for selected countries in 2023. The black solid line represents the frontier production function fitting curve; colored dotted lines show DALYs rate changes for individual countries. The black text indicates the 15 points with the largest distance differences; the blue text indicates the 5 countries with the smallest distance differences among low‐HDI countries; the red text indicates the 5 countries with the largest distance differences among high‐HDI countries.

The DEA‐derived frontier of minimum achievable ASDR conditional on HDI declined nonlinearly with development (Figure 4A). Most countries lay above the frontier (Figure 4B). Low‐HDI states (Afghanistan, Yemen, Pakistan) lay close to the frontier. Several upper‐HDI states departed substantially from the frontier: Kazakhstan’s ASDR exceeded the value predicted by its HDI, and the Gulf states (United Arab Emirates, Saudi Arabia, Qatar, Bahrain) recorded high prevalence and DALY burdens at high HDI levels (Figure 4B).

**FIGURE 5:**
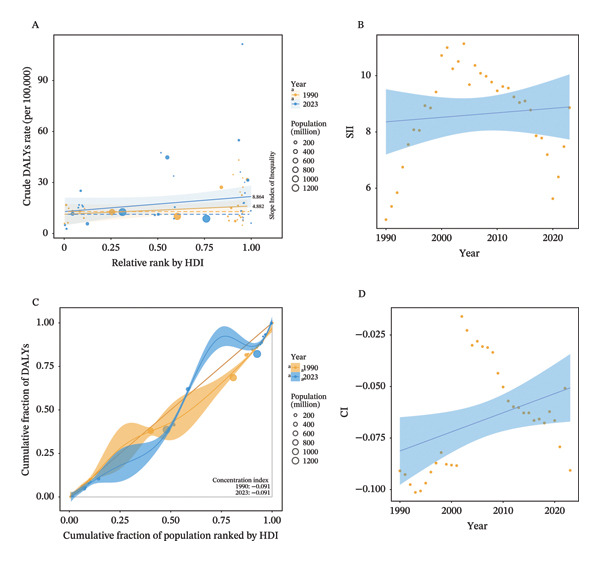
Health inequality analysis of NAFLD‐related DALYs rates, 1990–2023. (A) Trend of slope index of inequality for NAFLD‐related DALYs rates from 1990 to 2023. The slope index measures absolute inequality in NAFLD burden, while the concentration index measures relative inequality; larger absolute values indicate greater inequality. (B) Scatter plot of slope index of inequality for NAFLD‐related DALYs rates, 1990–2023. (C) Trend of concentration index for NAFLD‐related DALYs rates. (D) Scatter plot of concentration index for NAFLD‐related DALYs rates, 1990–2023.

Health‐inequality metrics evolved over the study period (Figure 5). The SII rose from 4.882 in 1990 to 8.864 in 2023, an 81.6% increase (Figure 5A, B). The SII trajectory was nonmonotonic, peaking at 11.137 in 2004, contracting to 5.629 during 2017–2020, then rising again. The CI was negative throughout the study period, ranging from −0.091 in 1990 to −0.091 in 2023; values rose to between −0.016 and −0.031 during 2002–2006 before returning to −0.091 by 2023 (Figure 5C, D).

### 3.5. Age, Sex, and Age‐Period‐Cohort Patterns

Age‐stratified absolute case counts peaked in middle adulthood (males approximately 42.88 million in the 35–39 band; females approximately 39.12 million in the 55–59 band) (Figure 6A); incident cases peaked in young adulthood (20–24 years) (Figure 6C); deaths and DALYs peaked at older ages (60–74 years) (Figure 6E, G). Rates rose monotonically with age for prevalence, mortality, and DALYs, while incidence showed a bimodal distribution with peaks at 20–24 and 65–69 years (Figure 6B, D, F, H). Males had higher prevalence rates than females across all age groups; female incidence exceeded male incidence after age 35; female mortality and DALY rates exceeded those of males after age 65.

FIGURE 6Age‐specific distribution characteristics of NAFLD disease burden in Asia. (A and B) Age‐specific prevalence rates and distribution of NAFLD. (C and D) Age‐specific incidence rates and distribution of NAFLD. (E and F) Age‐specific mortality rates and distribution of NAFLD. (G and H) Age‐specific DALYs rates and distribution of NAFLD.

**Figure:**
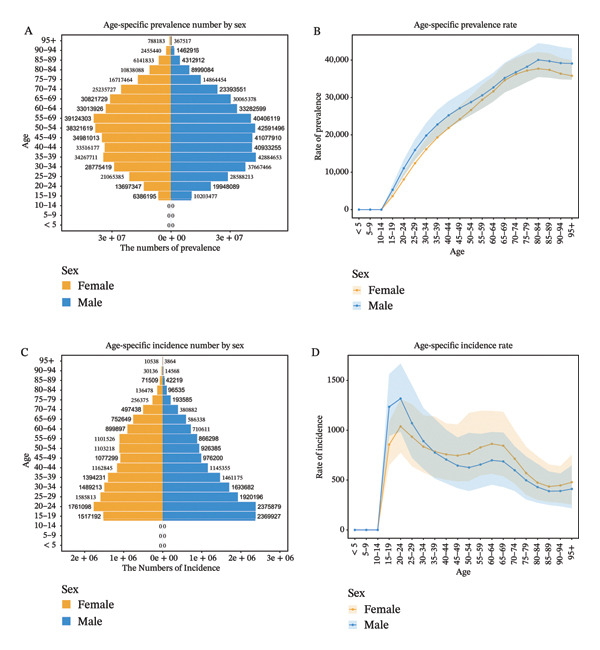

**Figure:**
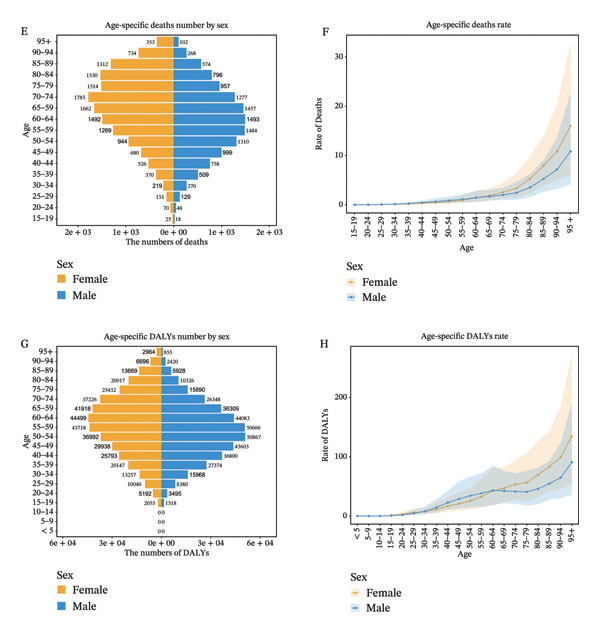

**FIGURE 7:**
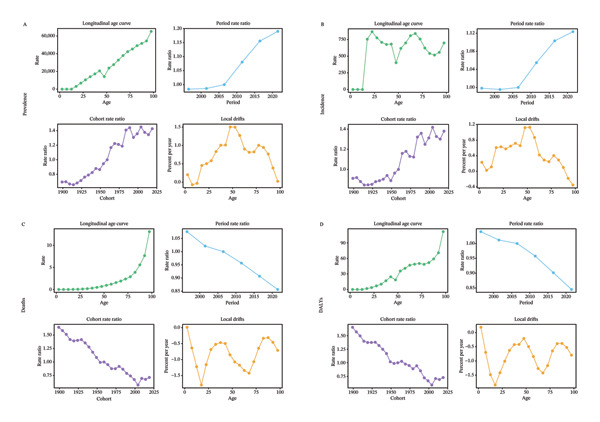
Age‐period‐cohort effect analysis of NAFLD in Asia (1990–2023). (A–D) Age‐period‐cohort effects for NAFLD prevalence, incidence, mortality, and DALYs, respectively. Top left: age effect plots showing the relative risk changes with age for each indicator. Top right: period effect plots with 2006 as a reference, displaying relative risks at different time points. Bottom left: cohort effect plots with the 1959 birth cohort as a reference, reflecting cumulative effects of lifestyle, environmental exposures, and other long‐term factors. Bottom right: local drift plots showing the annual percentage changes by age group, revealing dynamic trends in disease burden across different age populations.

Age‐period‐cohort analysis showed that the net of period and cohort effects, prevalence, and DALY age curves rose monotonically into very old age (Figure 7A, D). Period rate ratios (2006 referent) increased to 1.190 (prevalence) and 1.123 (incidence) by 2021 and declined to 0.857 (mortality) and 0.845 (DALYs) (Figure 7A–D, period subpanels). Cohort rate ratios (1959 referent) rose progressively for prevalence and incidence (CRR 0.695 and 0.911 in 1899 versus 1.427 and 1.380 in 2019) and fell progressively for mortality and DALYs (CRR 1.645 and 1.655 in 1899 versus 0.714 and 0.728 in 2019) (Figure 7A–D, cohort subpanels). Local drift was largest at the 47.5‐year age band for prevalence (+1.502%/yr) and at the 47.5–52.5‐year bands for incidence (Figure 7A, B, local‐drift subpanels).

### 3.6. Bayesian Projections to 2038

**FIGURE 8:**
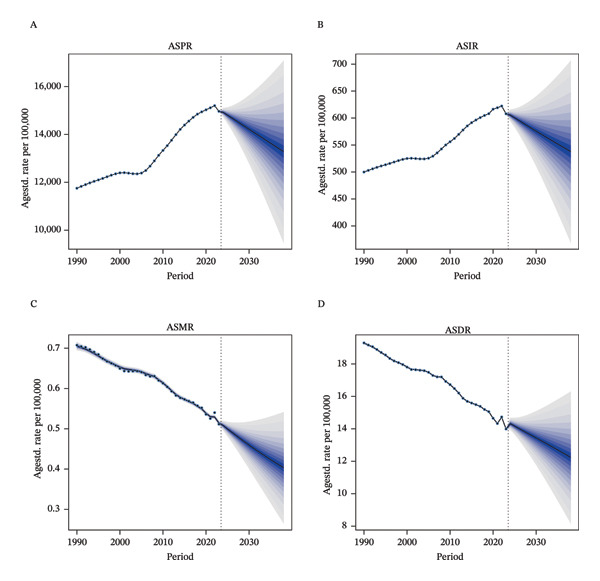
BAPC model‐based projections of age‐standardized prevalence (A), incidence (B), mortality (C), and DALYs (D) rates of NAFLD in Asia for 2024–2038.

BAPC projections from 2024 to 2038 indicated declines in all four indicators: ASPR from 14,919.851 to 13,285.897 per 100,000 (−10.96%) (Figure 8A); ASIR from 605.267 to 537.944 per 100,000 (−11.12%) (Figure 8B); ASMR from 0.508 to 0.404 per 100,000 (−20.47%) (Figure 8C); and ASDR from 14.317 to 12.244 per 100,000 (−14.48%) (Figure 8D). Predictive intervals widened progressively across the projection horizon (Figure 8A–D, shaded bands).

## 4. Discussion

Three findings of this analysis are, in our view, the principal contributions to Asia’s NAFLD literature, and we structure the discussion around them. The first is the morbidity–mortality divergence. The second is the demonstration that population‐level forces (growth and aging) dominate burden change, with East Asia constituting an important exception. The third is the documentation of a widening efficiency‐equity gap, which converts what would otherwise be a descriptive geography of disease into an actionable map of underperformance.

### 4.1. Mechanistic Interpretation of the Morbidity–Mortality Divergence

Rising ASPR and ASIR alongside falling ASMR and ASDR is the canonical signature of a chronic disease whose detection has improved faster than its incidence has stabilized, and whose case fatality has fallen faster than either. Three mechanisms are simultaneously at work in Asia. Detection has been transformed by the diffusion of transient elastography, controlled‐attenuation parameter, and MR‐based fat quantification, supplemented by validated serum panels (FIB‐4, NAFLD fibrosis score, ELF), with the most rapid uptake in East Asia’s tertiary networks [18–20]; this alone would produce apparent prevalence increases independent of any change in true incidence. Risk factors have continued to expand. Visceral adiposity, type 2 diabetes, dyslipidemia, and ultra‐processed food consumption have all risen across Asian populations [21–23], and air‐pollution and endocrine‐disrupting‐chemical exposures, which are plausible contributors documented in Chinese and South Asian cohorts [24], are superimposed on traditional metabolic risks. Prognosis has improved. Structured chronic‐liver‐disease pathways, hepatocellular‐carcinoma surveillance under semi‐annual ultrasound and AFP testing, expanded liver transplantation in tertiary centers, and the recent adoption of GLP‐1 receptor agonists with documented hepatic‐fat‐reduction effect have together compressed case fatality [25–27]. The implication for policy is that prevalence trends and mortality trends should not be aggregated into a single narrative. Reducing prevalence requires upstream metabolic action, while reducing mortality requires sustained clinical investment.

### 4.2. Why East Asia Is a Different Policy Problem

The decomposition result that population aging accounts for nearly half of East Asia’s 1990–2023 prevalence‐burden change is, to our knowledge, the highest such share documented for any major chronic‐disease analysis in the GBD‐Asia literature, and it has direct policy consequences. In East Asia, even effective strategies targeting population‐level metabolic risk—such as sugar‐sweetened beverage taxation, dietary interventions, and workplace physical‐activity programs—are likely to yield smaller absolute reductions than similar measures implemented in Southeast or South Asia, because population aging continues to enlarge the number of older adults already exposed to mid‐life metabolic risk [28]. East Asia’s historically large negative epidemiologic contribution to mortality (−338.71%) further indicates that the region is approaching the natural limits of clinical‐management gains and will increasingly need to address the prevalence side of the ledger. By contrast, in South and Southeast Asia, where population growth dominates, the highest‐yield investments are in primary prevention and access to early diagnosis, because rate‐level improvements operate on a still‐expanding base.

### 4.3. Efficiency Gaps as Accountability Metric

The DEA frontier reframes the policy conversation in an important way. Several countries with high ASDR are also low‐HDI states (Afghanistan, Yemen) for which expecting near‐frontier performance is unreasonable. Their ASDR sits close to the frontier, and they should not be the focus of NAFLD‐specific reform. Other countries with high ASDR are high‐HDI states whose performance is incommensurate with their resources. Kazakhstan is the most prominent example, and the Gulf states present a different mechanism [29]. Kazakhstan’s case is consistent with documented metabolic‐disease‐surveillance fragmentation, urban–rural healthcare imbalance, and a dietary‐westernization trajectory that has outpaced public‐health response. The Gulf pattern (high prevalence with relatively contained mortality but high DALY burden) reflects a population in which cardiometabolic risk has become near‐universal and in which intervention capacity exists but has not yet been mobilized at the population scale. The DEA framework allows ministries of health to distinguish between “we lack resources” and “we have resources but are not using them efficiently,” a distinction that ASMR alone cannot make.

### 4.4. Why Absolute and Relative Inequality Moved Differently

The simultaneous rise in SII and stability of the CI deserves comment because it reflects a structural feature of NAFLD distribution that is easy to miss. SII captures the absolute DALY‐rate gap between the lowest‐ and highest‐HDI populations; if both ends improve but the high‐HDI end improves faster, the absolute gap widens even as the relative shape remains constant. CI captures relative concentration; a stable CI means that, ranked by HDI, the share of total burden borne by lower‐HDI populations did not shift materially. The combination indicates that progress has been distributionally regressive in absolute terms, a pattern that is invisible if only one inequality metric is reported, and that should inform how regional health‐equity progress is described in policy briefings.

### 4.5. Implications for Clinical Practice and Policy

We translate findings into four pragmatic recommendations rather than a generic exhortation to act. First, NAFLD case finding should be embedded into existing diabetes and dyslipidemia care pathways, particularly in primary care [30]. The 35–59‐year window identified by APC analysis is when modifiable progression is most achievable, and it is also when most middle‐income Asian populations interact with screening services. Second, regional priorities should differ by burden architecture: Central Asia requires investment in clinical capacity and pharmacotherapy access; East Asia requires explicit planning for absolute‐burden growth driven by aging despite favorable rates; South Asia requires primary‐care infrastructure to identify cases at the inflection of expansion; Gulf states require population‐level metabolic intervention rather than further hospital‐based investment. Third, GLP‐1 receptor agonists, given their documented efficacy in reducing hepatic steatosis and weight [31, 32], are likely to materially affect future projections and should be incorporated into national essential‐medicine lists where evidence supports their use. Fourth, surveillance systems should report both absolute and relative inequality metrics on a recurring basis. Reporting only one risks declares progress where regression is occurring.

### 4.6. Limitations

#### 4.6.1. Limitations Inherent to GBD Modeling

All estimates reported here depend on DisMod‐MR 2.1’s integration of heterogeneous primary inputs and on the modeling assumptions required to enforce internal consistency between prevalence, incidence, remission, and excess mortality [33, 34]. Where vital registration is partial or hospital‐based ascertainment is incomplete, as in Afghanistan, Yemen, parts of Central Asia and Timor‐Leste, UIs will systematically underrepresent true uncertainty, because they capture only modeled variance and not unmeasured systemic bias. Country‐level estimates for low‐information settings should therefore be treated as plausible orders of magnitude rather than as point estimates, and intercountry comparisons that depend on small absolute differences should be interpreted accordingly.

#### 4.6.2. Disease‐Definition Heterogeneity

The transition from NAFLD to MAFLD (2020) and to MASLD (2023) [13] was incomplete during much of the observation window, and underlying primary studies used inconsistent ultrasound, transient‐elastography, biopsy, and serologic‐panel criteria. Although DisMod‐MR 2.1 mitigates these differences statistically, residual diagnostic heterogeneity probably contributes to between‐country variation, and likely biases recent‐period prevalence upward where elastography uptake has been steepest. We do not attempt to disentangle this empirically.

#### 4.6.3. Method‐Specific Limitations

Joinpoint regression assumes log‐linear segments and is sensitive to the maximum‐joinpoint specification; sensitivity analysis with three‐ and four‐joinpoint caps did not materially alter the substantive picture but is acknowledged. APC analysis cannot identify all three effects without an external constraint; we adopted the standard estimable‐function parameterization, which preserves identifiable contrasts but does not return absolute period or cohort levels. Das Gupta decomposition assumes additivity of the three factors and ignores potential interaction. DEA with HDI as the sole input is a one‐dimensional benchmarking that necessarily compresses healthcare system, dietary, and genetic determinants into a single score; multi‐input DEA is a useful direction for future work [35]. The BAPC framework embeds the assumption of trend continuation; structural breaks, including pandemic‐related care disruption and GLP‐1 RA diffusion, are not captured.

#### 4.6.4. What the Data Cannot Tell Us

The analysis is ecological and cannot test causal hypotheses about why specific countries deviate from the DEA frontier or why specific birth cohorts carry elevated risk. Mechanistic claims in the discussion are interpretive, drawing on the broader hepatology literature [36–38], and should be regarded as hypotheses generated by the data rather than tested against it. Linked individual‐level cohorts and randomized trials remain the appropriate vehicles for confirmation.

## 5. Conclusion

Asia’s NAFLD landscape between 1990 and 2023 is best characterized not as a uniform rise but as a transition. Morbidity is expanding, prognosis is improving, demographic forces dominate burden change everywhere except in aging East Asia, and both efficiency and equity gaps are widening rather than narrowing. The methodological combination used here (decomposition for attribution, DEA for benchmarking, joint SII and CI for equity, and BAPC for projection) produces an inferential picture that no single technique could yield, and we suggest it as a transferable template for regional burden science. For policy, the practical implication is that one‐size‐fits‐all continental strategies are likely to under‐deliver and that explicit, development‐stage‐specific responses, accompanied by accountability metrics that distinguish absolute from relative progress, are more likely to bend Asia’s NAFLD trajectory in the directions that matter most.

## Author Contributions

Xiaoheng Liu contributed to the conceptualization and design of the study, and acquired and curated the GBD data. Xiaoheng Liu and Chen Zhou performed the formal analysis and visualization. Xiaoheng Liu developed the methodology and conducted statistical modeling. Xiaoheng Liu wrote the original draft of the manuscript. All authors contributed to the review and editing of the manuscript. Xiaoheng Liu supervised the study and provided critical revisions.

## Funding

The authors received no financial support for the research, authorship, and/or publication of this article.

## Disclosure

An earlier version of this manuscript was deposited as a preprint on Research Square in 2025 [39]. The present submission has been substantially revised and rewritten in response to peer‐review feedback regarding text overlap; data, tables, and figures derive from the same underlying GBD 2023 extraction.

## Disclosure

All authors have read and approved the final manuscript.

## Ethics Statement

The authors have nothing to report.

## Conflicts of Interest

The authors declare no conflicts of interest.

## Data Availability

The data that support the findings of this study are available from the Global Burden of Disease (GBD) 2023 database (https://vizhub.healthdata.org/gbd-results/). All raw data analyzed during this study are publicly available via the GBD official website. Derived data supporting the conclusions of this article are available from the corresponding author upon reasonable request. All data generated or analyzed during this study are included in this published article.
